# Identification and evaluation of a thinning agent compatible with MegaCell DCS, an animal product-free corneal storage medium

**DOI:** 10.1007/s00417-012-2126-1

**Published:** 2012-08-12

**Authors:** Valerie A. Smith, Terrell K. Johnson

**Affiliations:** 1 Academic Unit of Ophthalmology, University of Bristol, Bristol Eye Hospital, Lower Maudlin Street, Bristol, BS1 2LX UK; 2 SAFC, PO Box 14508, St Louis, MO 63178 USA

**Keywords:** Cornea, Corneal organ culture medium, Corneal transplant, Corneal endothelium, Epithelial cells, Keratocytes

## Abstract

**Purpose:**

MegaCell DCS, an animal product-free culture medium formulated for storing corneas, is superior to the traditionally used MEM (Eagle’s) with Earles salts, Hepes, and supplemented with foetal calf serum (2 %), glutamine and an antibiotic cocktail (EB MEM). Because this medium does not prevent corneal swelling, and Dextran T500, which is traditionally used for reversing this process before transplant may have adverse effects on corneas, the purpose of the current investigation was to identify an alternative polymer that is compatible with MegaCell DCS.

**Methods:**

Corneas maintained in MegaCell DCS or EB MEM were transferred to either EB MEM 5 % Dextran T500 or MegaCell DCS containing 5 % Dextran T500, 4 % polyethylene glycol (PEG) 10,000, PEG 35,000 (2 %, 3 %, 4 %) or Poloxamer 188 (4 %). Endothelial cell losses were determined and corneal hydration levels measured. Stromal cell cultures were generated and immunostained with anti α-SMA antibody. Janus Green was used to compare the viability of endothelial cells of corneas maintained in MegaCell DCS and EB MEM and respectively thinned with PEG 35,000 and Dextran T500.

**Results:**

The rates of endothelial cell loss from corneas held in MegaCell DCS and thinned in MegaCell DCS containing 5 % Dextran T500, 4 % PEG 10,000 and 4 % Poloxamer 188 for 6 days were similar. When explants of these corneas were cultured myofibroblasts were generated. Although at concentrations of 4 % (w/v) both PEG 10,000 and Poloxamer 188 caused excessive dehydration, the hydration levels of corneas held in MegaCell DCS containing 3 % PEG 35,000 were similar to those of corneas held in EB MEM 5 % Dextran T500. Endothelial cell losses after 6 days were negligible, explants of the corneas generated uniform fibroblastic stromal cell cultures and the extents of Janus Green staining were similar. Over 20 days the inclusion of 5 % Dextran T500 in EB MEM but not 3 % PEG 35,000 in MegaCell DCS, increased the rate of endothelial cell loss.

**Conclusion:**

PEG 35,000 at a concentration of 3 % w/v does not induce endothelial cell loss and is compatible with MegaCell DCS for thinning corneas prior to transplantation.

## Introduction

The primary function of corneal endothelia is to passively transport nutrients and solutes from the aqueous humour to the more superficial layers, and simultaneously actively pump water from the stroma into the aqueous. Although these activities are coordinated in vivo to maintain the slightly dehydrated state required for optical transparency, in culture corneas swell, possibly as a result of the shutting down of their endothelial cell Na^+^/K^+^ ATPase pumps and the loss of several layers of epithelium. For this reason, to facilitate suturing and post-operative recovery, almost all organ cultured corneas stored in European Eye Banks are thinned in medium containing the polyglucose polymer, Dextran T500, prior to transplant [1, 2].

Despite the widespread use of Dextran T500, there are reservations over its toxicity. Initial observations of Van der Want and Pels [3], showing that corneas placed in medium containing Dextran T500 for 7 days accumulated dextran in almost all cellular components, have been corroborated by Redbrake et al. [4]. In addition to observing both intra- and extracellular deposits throughout the cornea, these authors noted that intracellular dextran accumulated after only 1 day and a significant amount of extracellular dextran was insoluble. Other authors have reported that concomitant with the accumulation of dextran, the loss of endothelial cells increased significantly from a base level of 0.9 % per day in dextran-free medium to 15 % over a period of 2 days in media containing dextran [5, 6]. The current consensus of opinion is that corneas destined for transplant should be left no longer than 4 days in Dextran T500 [7, 8].

Recently we investigated the suitability of serum-free MegaCell™MEM and an animal product-free medium (MegaCell DCS) for storing corneas. Relative to EB MEM [Minimum Essential Medium (Eagle’s) with Earles salts, 25 mM Hepes, supplemented with glutamine (20 mM) and foetal calf serum (2 % v/v)] which is widely used for storing corneas in European Eye Banks [9], these media prolonged the viability of corneal endothelial cells and improved their morphological appearance [10, 11]. The purpose of this study was to identify and evaluate a thinning agent, other than Dextran T500 that does not compromise the viability of the corneal endothelium and is compatible with MegaCell DCS.

## Materials and methods

### Experimental material

The pairs of corneas used in this investigation had been granted research permission by the families of the donors and are listed in Tables 1 and 2. They were obtained from the Bristol CTS Eye Bank and considered unsuitable for transplantation because of medical contraindications. The work undertaken adhered to the tenets of the declaration of Helsinki and had NHS Research Ethics approval.

**Table 1:** Characteristics of the corneas used to evaluate endothelial cell loss after thinning

Cornea	Age (years)/Sex	Preincubation time (days)	Initial ECDs (cells/mm^2^)
1A/B	82 M	9	1690; 1700
2A/B	69 M	11	2160; 2070
3A/B	66 M	15	2330; 2190
4A/B	86 M	12	1300; 1340
5A/B	46 M	11	2390; 2300
6A/B	33 F	10	2510; 2480
7A/B	81 M	8	1980; 2570
8A/B	41 M	12	1570; 1740
9A/B	91 F	12	2360; 1920
10A/B	79 F	8	2250; 2510
11A/B	74 M	7	2570; 2680
12A/B	84 F	2	1420; 1500
13A/B	75 M	4	2710; 2890
14A/B	58 M	13	2300; 2300
15A/B	65 M	13	2120; 2010
16A/B	92 M	1	2240; 2150
17A/B	41 F	5	2680; 2790
18A/B	82 M	11	2630; 2630
19A/B	72 F	9	2420; 2300
20A/B	80 M	10	2450; 2450
21A/B	88 M	1	2890; 2800
22A/B	38 F	7	2540; 2650
23A/B	71 M	4	2420; 2450
24A/B	89 F	4	2590; 2850
25A/B	82 M	10	2095; 1980
26A/B	83 M	10	2150; 2070
27A/B	90 M	9	2390; 2480
28A/B	59 F	9	2830; 2800
29A/B	90 M	14	2960; 2660
30A/B	81 M	14	2610; 2700
31A/B	64 F	10	2520; 2420
32A/B	81 M	8	3020; 3060
33A/B	84 F	12	2000; 2060
34A/B	62 M	10	2720; 2610
35A/B	86 M	11	2600; 2290
36A/B	82 M	11	2240; 2030
37A/B	83 F	15	2420; 2510
38A/B	74 F	13	2850; 2790
39A/B	81 F	11	2540; 2610
40A/B	73 F	11	2690; 2630
41A/B	60 M	13	2500; 2330
42A/B	52 M	11	2090; 2150
43A/B	69 F	13	2240; 2190
44A/B	53 M	12	2240; 2190
45A/B	73 M	9	2235; 2130
46A/B	59 M	8	2030; 2090
47A/B	68 M	8	2060; 2450
48A/B	55 F	13	2510; 2490

**Table 2:** Corneas used for investigating endothelial cell survival after storing in EB MEM and thinning with 5 % Dextran T500 (**a**) or in MegaCell DCS and thinning with 3 % PEG 35,000 (**b**)

Cornea ID	Age (years)/sex	Preculture (days)	In EB MEM MC DCS (days)	Initial ECDs (cells/mm2)
1A/B	86 F	16	35	2090/1885
2A/B	72 M	13	32	2540/2580
3A/B	83 F	10	40	2582/2500
4A/B	33 M	0	40	2376/2418
5A/B	74 F	8	32	2090/1885
6A/B	82 M	8	35	2418/2295
7A/B	86 F	0	39	2746/2705
8A/B	49 F	5	39	2499/2622
9A/B	66 F	14	32	1558/1925
10A/B	81 F	11	32	2335/2663
11A/B	82 M	0	49	2048/2008
12A/B	64 F	0	45	2008/2090
13A/B	41 M	0	47	2171/2211
14A/B	71 F	19	27	2520/2550
15A/B	95 M	16	27	1804/1925
16A/B	75 M	11	27	1970/2008
17A/B	76 F	0	44	2598/2662
18A/B	53 F	28	22	2458/2376
19A/B	63 M	20	27	1722/1683
20A/B	67 M	15	27	2173/2213
21A/B	78 M	11	27	1639/1639
22A/B	66 M	12	22	1232/1188
23A/B	82 F	7	42	2300/1970
24A/B	45 M	4	35	2170/2704

### Corneal storage

Corneas held under standard eye banking conditions in EB MEM [MEM containing Earles salts, Hepes (25 mM), and supplemented with glutamine (Invitrogen Ltd), a penicillin, streptomycin and amphotericin cocktail (Sigma-Aldrich Ltd) and 2 % foetal calf serum (FCS, Invitrogen Ltd)], were transferred to 100 ml din bottles containing either fresh EB MEM, MegaCell™MEM or MegaCell DCS supplemented only with the antibiotic cocktail. After incubating for a minimal period of 3 weeks the corneas were suspended in 60 ml din bottles containing either EB MEM 5 % Dextran T500 or MegaCell DCS containing 5 % Dextran T500, PEG 10,000 (4 %), PEG 35,000 (2 %, 3 %, 4 %) or Poloxamer 188 (4 %) for 6 days unless otherwise specified.

### Corneal endothelial cell density and rate of loss measurement

As described previously [10], the corneal endothelia were examined before and during the thinning process under hypotonic sucrose (1.8 % w/v) after staining with trypan blue (0.4 % w/v, Sigma-Aldrich Ltd). Photographs were taken every other day. From these, endothelial cell densities were estimated and plotted against time to determine relative rates of cell loss.

### Corneal hydration estimation

Discs (8 mm) were trephined from the centres of the thinned corneas, blotted dry and placed in small, preweighed petri dishes. They were then weighed and reweighed after drying at 60 °C to constant weight. Percentage hydration levels were calculated from the formula (wet weight – dry weight/wet weight) x 100.

### Corneal cell culture

Explants were prepared from corneas that had been stored in MegaCell DCS and thinned for 6 days in EB MEM Dextran T500 or MegaCell DCS containing Dextran T500, PEG 10,000, PEG 35,000 or Poloxamer 188. They were placed in either 25 ml flasks or 6 well plates and incubated under 5 % CO_2_/95 % air at 36 °C in MegaCell™ MEM containing the antibiotic antimycotic mix and FCS (5 %). After 7 days, the media was replenished every 3–4 days.

### Anti α-SMA antibody immunostaining

Cultures of corneal stromal cells were washed twice with PBS, fixed with formol saline (10 % v/v) for 20 min at room temperature, permeabilised with Triton X-100 (0.25 % v/v) and again rinsed with PBS. They were then sequentially incubated for 1 h at 36 °C with a mouse anti-human actin [alpha smooth muscle isoform] monoclonal antibody (Millipore Ltd) diluted 1:500 in PBS containing FCS (5 % v/v) and a 1:1000 diluted rhodamine conjugated goat anti-mouse IgG (Millipore Ltd), The washing solution was PBS/Tween 20 (0.1 %). After a final rinse in PBS, the cultures were photographed under a Leitz Dialux 22 EB fluorescence microscope. Primary antibody was omitted from cultures used as controls.

### Janus Green staining

A method using Janus Green as a vital stain for assessing the extent of endothelial cell damage has been reported [12, 13]. For this study, Janus Green B (Sigma Aldrich Ltd) was dissolved at a concentration of 1 mg ml^-1^ in isotonic saline and filter sterilised. All corneas, with their scleral rim intact, were placed in small petri dishes, endothelial cell side up. These were covered with the Janus Green B solution (150 μl) for 5 min before extensively rinsing the corneas with fresh saline. Subsequently 8 mm buttons were trephined from the centre of each cornea, placed in 48 well plates and covered with absolute ethanol (500 μl) to extract the retained dye. Aliquots of the ethanol (2 × 200 μl) were then removed and placed in 96 well plates. The optical densities of these solutions were measured at 654 nm in a Spectromax spectrophotometer. Corneas of predetermined endothelial cell density were treated with sodium azide (0.1 M in isotonic saline for 16 h) to serve as controls.

### Statistical analyses

Data are expressed as mean ± SD. Rate equations for endothelial cell losses were obtained by regression analysis. The 2-tailed Student’s *t* test for paired data was used to assess correlative significance.

## Results

Hydration levels of and endothelial cell losses from corneas thinned in MegaCell™MEM with Dextran T500 (5%w/v) and PEG 10,000 (4 % w/v)

For corneas stored in EB MEM, the extent of deswelling in 5 % Dextran T500 is achieved using 4 % PEG 10,000 [14]. In this investigation, preliminary experiments indicated that the hydration levels of 12 pairs of corneas (corneas 1–12, Table 1) stored for 3 weeks in MegaCell™MEM before transferring to MegaCell™MEM containing either 5 % w/v Dextran T500 or 4 % PEG 10,000 for 14 days were 84.4 ± 1.6 % and 85.0 ± 2.3 % respectively and not significantly different (p-value 0.67). Although calculated rates of endothelial cell loss from these corneas, respectively 38.3 ± 22.43 and 26.0 ± 9.4 cells per day over 12 days, were also statistically similar (p-value 0.12), beyond 14 days the endothelia of the 5 % Dextran T500 thinned corneas did not survive whereas those of corneas thinned with 4 % PEG 10,000 remained morphologically unchanged.

### Endothelial cell loss from corneas thinned in MegaCell™DCS with Dextran T500 (5%w/v), PEG 10,000 (4 % w/v) and Poloxamer 188 (4 % w/v)

Corneas 13–18 (Table 1) that had been maintained in MegaCell DCS for 3 weeks were transferred to either MegaCell DCS containing Dextran T500 or MegaCell DCS containing 4 % PEG 10,000 for a period of 6 days. The initial estimated endothelial cell densities were 2447 ± 256 and 2462 ± 360 cells mm^-2^ respectively and not significantly different (p-value 0.76). Over the following 6 days the estimated rate of cell loss from the corneas held in MegaCell DCS containing 5 % Dextran T500 was negligible and from the corneas held in MegaCell DCS containing 4 % PEG 10,000, 15 cells mm^-2^ per day. The latter set of corneas subsequently thickened and counting their endothelial cells became increasingly problematical.

Similar experiments were performed using corneas 19–24 (Table 1) that after incubation in MegaCell DCS were transferred to either MegaCell DCS containing 4 % PEG 10,000 or MegaCell DCS containing 4 % Poloxamer 188. On this occasion the initial ECDs were 2552 ± 179 and 2583 ± 218 cells mm^2^ and the respective rates of endothelial cell losses were 45.5 ± 11.8 and 55.3 ± 26.6 cells per day (p-value 0.48), but again by day 6 all corneas were thickening.

### Comparative hydration levels of corneas thinned in EB MEM 5 % Dextran T500 and in MegaCell DCS containing 5 % Dextran T500, 4 % PEG 10,000 or 4 % Poloxamer P188

Estimated hydration levels of tissue trefined from the centre of corneas thinned in EB MEM 5 % Dextran T500 or MegaCell DCS containing 5 % Dextran T500, 4 % PEG 10,000 or 4 % Poloxamer P188 are given in Table 3. These data indicate that in MegaCell DCS, the thinning capacities of PEG 10,000 and Poloxamer P188, at concentrations of 4 % w/v, are greater than 5 % w/v Dextran T500. They may also account for the observed time dependent corneal thickening.

**Table 3:** Estimated hydration levels of corneas stored in MegaCell DCS and thinned in either EB MEM, 5 % Dextran T500, MegaCell DCS, 4 % PEG 10,000 or MegaCell DCS 4 % Poloxamer 188

Thinning Medium	Number of corneas	Estimated final hydration (%)
EB MEM Dextran T500	5	85.94 ± 1.14
MegaCell DCS, Dextran T500	5	84.90 ± 0.97
MegaCell DCS, PEG 10,000	7	80.71 ± 2.91
MegaCell DCS, Poloxamer 188	7	80.21 ± 3.18

### Effect of Dextran T500, PEG 10,000 and Poloxamer P188 on the morphology of stromal cells cultured from corneas held in MegaCell DCS

**Fig. 1:**
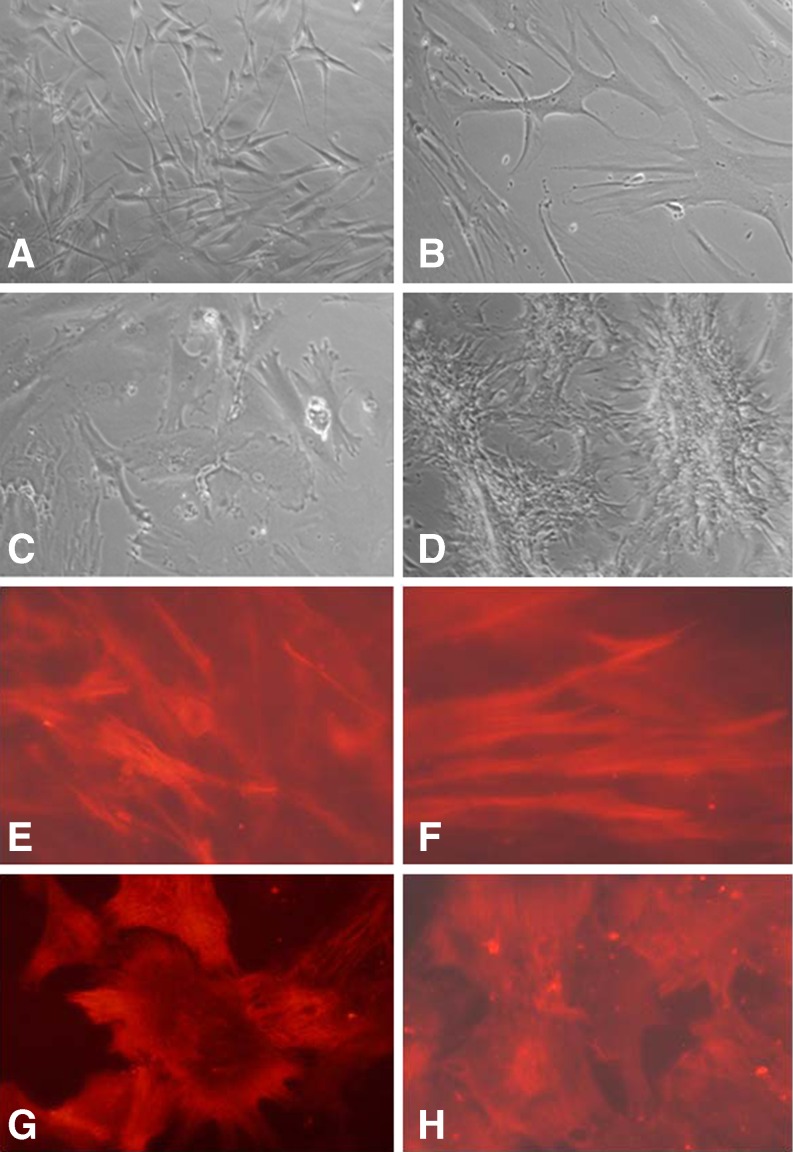
Morphological variation and anti-SMA antibody staining of stromal cell outgrowths from corneas held in EB MEM 5 % Dextran T500 (**a**, **e**), MegaCell DCS 5 % Dextran T500 (**b**, **f**), MegaCell DCS 4 % PEG 10,000 (**c**, **g**), MegaCell DCS 4 % Poloxamer 188 (**d**, **h**) for 6 days. Original mag x200

Previous work showed that explants prepared from corneas held in MegaCell DCS readily generated both epithelial and phenotypically uniform, fibroblastic stromal cell cultures if FCS were added to their incubation medium [11]. Although this would usually initiate after 1 week, for corneas that had been thinned, epithelial cell outgrowths were not observed and stromal cell outgrowth, when and if it occurred, frequently initiated as late as 4 weeks after culturing the explants. The morphology of these cells was extremely varied but not necessarily dictated by which thinning reagent had been used. Examples are shown in Fig. 1a–d. Confirmation that myofibril differentiation had been induced in many of the cultures as a result of thinning the corneas was obtained by immunostaining some of the stromal cell cultures with anti α-SMA antibody. Representative staining patterns are shown in Fig. 1e–h.

### Effect of PEG 35,000 concentration on corneal endothelial cell density, morphology and hydration levels

The observation that MegaCell DCS medium containing Dextran T500 (5 % w/v), PEG 10,000 (4 % w/v) or Poloxamer 188 (4 % w/v) induced myofibril formation suggested that the keratocytes of corneas incubated in these media may become activated as a result of excessive dehydration.

**Fig. 2:**
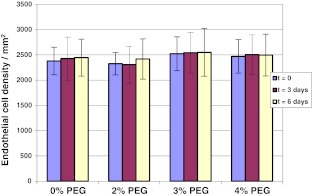
Endothelial cell densities of corneas held in MegaCell DCS containing 2 %, 3 % and 4 % w/v PEG 35,000 for 6 days

**Fig. 3:**
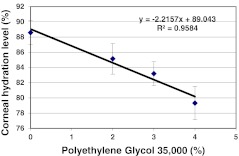
Estimated hydration levels of corneas held in MegaCell DCS containing 2 %, 3 % and 4 % w/v PEG 35,000 for 6 days

**Fig. 4:**
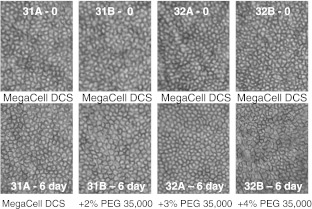
Morphology of the endothelia of corneas (pairs 31 and 32, Table 1) after incubation in MegaCell DCS for 3 weeks (*top row*) and in MegaCell DCS and MegaCell DCS containing 2 %, 3 % and 4 % w/v PEG 35,000 for 6 days (*bottom row*)

To address this possibility, corneal pairs 25–48 (Table 1) that had been maintained in MegaCell DCS for 3 weeks were transferred to fresh MegaCell DCS containing either 0, 2 %, 3 % or 4 % PEG 35,000. Over a period of 6 days the endothelia of these corneas was examined microscopically and their cell densities determined. Subsequently 8 mm buttons were trefined from the corneal centres for estimating corneal hydration levels and cell cultures were generated from the surrounding corneal tissue. The results presented in Figs 2 and 3 respectively indicated that the inclusion of PEG 35,000 in MegaCell DCS did not induce endothelial cell loss and confirmed that the hydration level achieved in MegaCell DCS containing 4 % PEG 35,000 was similar to that achieved in 4 % PEG 10,000, significantly lower than that of corneas incubated in EB MEM containing 5 % Dextran T500. Representative photographs of the endothelia of corneas incubated in MegaCell DCS containing 0, 2 %, 3 % or 4 % PEG 35,000 for 6 days are shown in Fig. 4.

### Cell growth from corneas held in MegaCell DCS and thinned in EB MEM 5 % Dextran T500 or MegaCell DCS containing 2 %, 3 % and 4 % PEG 35,000

**Fig. 5:**
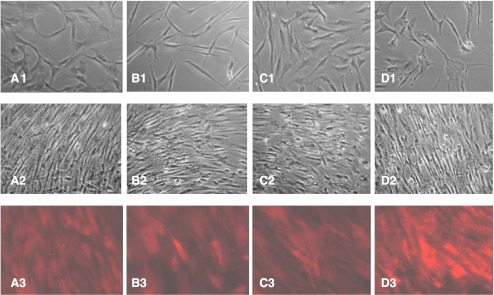
Stromal cell cultures derived from corneas held in MegaCell DCS for 3 weeks then EB MEM 5 % Dextran T500 (**a**), MegaCell DCS 5 % Dextran T500 (**b**), MegaCell DCS 3 % PEG (**c**) and MegaCell DCS 4 % PEG (**d**) for 6 days. Row 1–10 days after culturing explants. Row 2 - When confluent. Row 3 - When confluent and immunostained with anti-SMA antibody. Original mag x200

Explants of corneas incubated in Megacell DCS and MegaCell DCS containing 2 % PEG 35,000 produced confluent layers of epithelial cells (not shown) 10 days after culturing. By contrast, explants of the corneas thinned in EB MEM 5 % Dextran T500 and MegaCell DCS containing 3 % or 4 % PEG 35,000 produced only stromal cells. While those of the corneas thinned with EB MEM 5 % Dextran T500 and 3 % PEG 35,000 appeared to be fibroblasts, significant numbers of those from corneas thinned with 4 % PEG 35,000 were phenotypically myofibroblastic. Although when confluent these cultures became morphologically similar, when probed with anti α-SMA antibody, the level of staining in cultures from corneas thinned with 4 % PEG 35,000 was significantly more intense than in those thinned with Dextran T500 or 3 % PEG 35,000 (Fig. 5).

### Endothelial cell losses from corneas incubated in MegaCell DCS and EB MEM and respectively thinned in MegaCell DCS with PEG 35,000 (3 % w/v) or Dextran T500 (5%w/v)

**Fig. 6:**
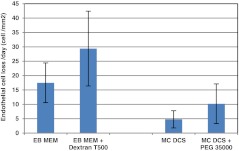
Rates of endothelial cell loss from corneas maintained in EB MEM and MegaCell DCS and respectively thinned with 5 % Dextran T500 and 3 % PEG 35,000

**Fig. 7:**
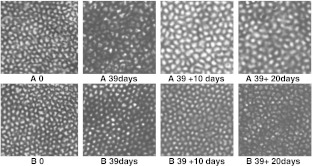
Endothelial morphology of corneas (pair 7, Table 2) maintained in EB MEM (**a**) and MegaCell DCS (**b**) over 39 days and respectively thinned with 5 % Dextran T500 and 3 % PEG 35,000 for 10 and 20 days

To compare directly the dependence of corneal viability on culture medium composition, paired corneas 1–10 (Table 2) were incubated in either MegaCell DCS or EB MEM for periods ranging from 32 to 40 days in addition to the preincubation period in EB MEM before transferring to either MegaCell DCS containing PEG 35000 (3 %) or EB MEM containing Dextran T500 for time periods exceeding 20 days. The estimated rates of endothelial cell losses from these corneas during storage and in response to the inclusion of PEG 35,000 (3 %) or Dextran T500 (5 %) are given in Fig. 6. These data show that fewer endothelial cells were lost from the corneas held in MegaCell DCS than from corneas held in EB MEM (p value < 0.001) and, as observed previously [11], that the preincubation time effects endothelial cell loss from the latter but not the former set of corneas (p values 0.002 and 0.53 respectively). They also indicate that for corneas incubated in MegaCell DCS, the inclusion of PEG 35,000 for 20 days has no significant effect upon endothelial cell loss (p value = 0.076) whereas for corneas incubated in EB MEM the inclusion of Dextran T500 increases the rate of endothelial cell loss (p value = 0.018). Photographs of the endothelia of corneas (pair 7, Table 2) that had not been preincubated, are shown in Fig. 7.

### Janus Green staining

Two additional sets of paired corneas (11–17 and 18–24, Table 2) were incubated in MegaCell DCS and EB MEM for a total period of 5–6 weeks before transferring to MegaCell DCS containing PEG 35,000 (3 %) or EB MEM containing Dextran T500 (5 %) for 6 days.

The endothelial cell densities of these corneas were monitored. Again the calculated endothelial cell losses (Table 4) were significantly less in MegaCell DCS and MegaCell DCS/PEG 35,000 than in EB MEM and EB MEM/Dextran T500. The results also indicated that over a thinning period of 6 days the inclusion of PEG 35,000 or Dextran T500 did not affect significantly the rates of endothelial cell loss in these media (p values 0.22 and 0.30 respectively). The corneas were then stained with Janus Green, together with others for which their endothelial cell densities had been estimated before placing them in the isotonic saline containing sodium azide.

**Table 4:** Rates of endothelial cell loss from corneas maintained in EB MEM (A) or MegaCell DCS (B) and thinned respectively with Dextran T500 and PEG 35,000

Corneas	Endothelial Cell Losses (cells/day) Maintenance Thinning
Set 1 (n = 7)	
A corneas	25.4 ± 8.7 29.7 ± 18.6
B corneas	7.0 ± 4.9 5.3 ± 4.2
Set 2 (n = 7)	
A corneas	22.2 ± 11.2 36.6 ± 38.8
B corneas	10.0 ± 10.0 17.4 ± 10.2

Visually, after staining, the endothelia of the azide treated corneas were almost uniformly dyed whereas on corneas thinned in MegaCell DCS/PEG 35,000 and EB MEM/Dextran T500, a few lines of dye tracked radially inwards from the limbus but generally remained in the peripheral region.

**Fig. 8:**
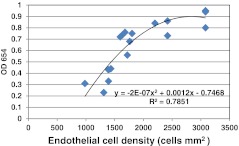
Relationship between Janus Green dye uptake and the ECD of corneas subsequently treated with 0.1 M sodium azide

The best fit relationship between endothelial cell density and OD_654_ of the Janus Green dye extracted after killing these cells with sodium azide was polynomial (Fig. 8) and the levels of staining on the MegaCell DCS/PEG 35,000 and EB MEM/Dextran T500 thinned corneas were 0.15 ± 0.03 and 0.16 ± 0.06 respectively and statistically similar (p value 0.47). Although it was not possible to relate these readings to the densities of dead endothelial cells because they fell well below the calibration limits, given the staining patterns it was considered likely that the stain picked out areas of cell loss/exposed Descemets membrane [13].

## Discussion

MEM with Earles salts, Hepes and supplemented with glutamine, antibiotics and foetal calf serum (EB MEM) was the culture medium originally chosen to store corneas. Together with the Dextran T500 used for thinning the corneas prior to transplantation it is still widely used in European Eye Banks.

Although EB MEM is adequate for the purpose of storing corneas, the failures of this holding system over prolonged periods of time relate to the fact that the medium itself is of poor buffering capacity and, additionally, unless the added FCS concentration is increased from the traditionally used 2 % to around 10 %, the corneas deteriorate significantly after 3 weeks in culture [15]. This measure, however, ceased to be an option since the outbreak of bovine spongiform encephalopathy (BSE) in the UK and regulatory bodies desired the use of media that do not require the addition of serum to sustain the tissue.

In response to this, MegaCell DCS was formulated for storing corneas prior to transplantation. In contrast to the fate of corneas maintained in EB MEM, those maintained in MegaCell DCS do not deteriorate after 3 weeks in culture. Furthermore, although this animal product-free medium does not support cell proliferation, after prolonged storage periods, corneal epithelial and stromal cells remain viable and will proliferate once foetal calf serum has been added to the medium [11].

The use of MegaCell DCS does not prevent corneas swelling when cultured and hence preclude the necessity of reversing this process prior to transplant.

The practice of using high molecular weight polysaccharides for dehydrating tissues is well understood and Dextran T500 became the polymer of choice for thinning organ cultured corneas prior to transplant because, with a molecular weight of 500,000, it would not be expected to penetrate tissues and cells. Despite this it does [3–5] and the reason probably relates to a high level of polydispersity [16]. For this reason and because solutions of Dextran T500 are not easy to prepare, an alternative for using with MegaCell DCS was sought.

Preliminary experiments carried out using corneas held in MegaCell™MEM and transferred to the same medium containing 4 % PEG 10,000 indicated that this polymer would thin them with minimal endothelial cell loss over a 14 day period. However, when this culture medium was replaced with MegaCell DCS problems, unrelated to endothelial viability as judged by the lack of trypan blue staining, became apparent. The polymers initially included in the MegaCell DCS were Dextran T500 (5 %), PEG 10,000 (4 %) and Poloxamer 188 (4 %) which is considered better than Dextran T500 for deswelling corneas [17], has been patented for this usage [6] and is one of a series of synthetic amphiphilic aqueous gels, marketed as Pluronics (BASF Chemical Company Ltd) and patented for use in the Pharmaceutical Industry in 1973. In all cases the corneas became microscopically opaque after 6 days, were excessively dehydrated and difficult to generate cell cultures from. Stromal cells that eventually grew out of explants were of mixed morphology and included myofibroblasts.

In culture, corneal stromal cells treated with TGF-β differentiate into myofibroblasts [18, 19]. In vivo, the transformation of fibroblasts into myofibroblasts is a known consequence of trauma, associated with corneal haze and linked to wound healing [20, 21]. Given that explants of corneas maintained for prolonged periods in MegaCell DCS readily produce homogeneous cultures of fibroblastic stromal cells, keratocyte activation in corneas subsequently thinned for 6 days with 5 % Dextran T500, 4 % PEG or 4 % Poloxamer 188 possibly relates to excessive dehydration. When Poloxamer 188 was judged to be superior to Dextran T500 for deswelling corneas in an animal compound-free medium [6] its concentration was not given. Subsequently, when comparing the toxicities of a number of poloxamers in CorneaMax (Eurobio, Les Ulys, France), the stated concentration of Poloxamer 188, used to achieve a standardised osmolality, was 3 % w/v [17].

In addition to over-dehydration, however, polymer uptake may also contribute to changes in keratocyte physiology. That this is possible relates to observations that explants of corneas incubated in EB MEM with 5 % Dextran T500 also produce myofibroblasts. Additionally, the polydispersity of PEG is significantly less than Dextran T500 and although corneas thinned with 4 % PEG 10,000 became microscopically opaque after 6 days, those thinned with 4 % PEG 35,000 remained clear.

Retrospectively it was realised that the influence of MegaCell DCS over the corneal thinning process possibly related to the fact that it contains 4.5x more glucose than MegaCell MEM or EB MEM. In investigating this, it was found that the hydration level achieved using 5 % Dextran T500 in EB MEM was matched by concentrations of 3 % PEG 35,000 in MegaCell DCS and significantly less in MegaCell DCS containing 4 % PEG 35,000. In addition, when confluent, stromal cell cultures prepared from corneas thinned in 4 % PEG 35,000 retained high levels of anti α-SMA antibody staining relative to those thinned in Dextran T500 and 3 % PEG 35,000. This observation indicates that there could be post-transplantation consequences to the metabolic status of stromal cells: Myofibroblasts present in grafted corneas may undergo IL-1 induced apoptosis [18], but if persistent, secrete the complement of collagens and proteoglycans characteristic of scar tissue and cause corneal haze [20, 22].

Polyethylene glycol, used at a concentration of 3 %(w/v) in MegaCell DCS was, therefore, considered to be the polymer of choice for thinning corneas: In contrast to the corneas thinned with Dextran T500, even after 20 days in the presence of this reagent they appeared healthy, their endothelial cell mosaic was morphologically regular and there was little reduction in density. However, to confirm that these cells remained viable, Janus Green was employed. This is a vital dye that is soluble in saline, binds nuclear and cytoplasmic components of dead cells and once bound can be released from the tissue with ethanol. Although unsuitable for checking the suitability of corneas destined for transplantation it has been used to assess corneal endothelium viability for experimental purposes: Because both dead endothelial cells and Descemet’s membrane in areas of cell loss are equally stained [13], the overall extent of damage can be determined.

In an attempt to use Janus Green to quantify the numbers of dead endothelial cells on corneas that had been either maintained in MegaCell DCS and thinned in MegaCell DCS containing PEG 35,000 or maintained in EB MEM and thinned in EB MEM containing Dextran T500, it was found that there was a polynomial relationship between dead endothelial cell density and the optical density of the ethanolic solution of dye extracted from the tissue. It was also found that the extent of staining on the endothelia of the corneas thinned in MegaCell DCS/PEG 35,000 and EB MEM/Dextran T500 for 6 days was similar. Visual examination of the corneas after staining indicated that the dye was primarily located in folds, tracking inwards from the limbus. Thus, although the area of endothelial damage may be the same on both sets of corneas, the numbers of viable endothelial cells remaining on the corneas maintained in MegaCell DCS and thinned in MegaCell DCS/PEG 35,000 were significantly higher than those on the corneas maintained in EB MEM and thinned in EB MEM/Dextran T500.

In summary, PEG 35,000 included at a concentration of 3 % w/v, is the polymer of choice for returning corneas maintained in MegaCell DCS to physiological hydration levels prior to transplantation. It is non toxic to the corneal endothelium and does not induce corneal endothelial cell loss.
